# Evidence summary for the management of sleep disorders after malignant tumor surgery

**DOI:** 10.3389/fneur.2025.1580216

**Published:** 2025-06-13

**Authors:** Chang Yang, Yuanfei Liu, Bang Xiao, Jing Yang, Qin Zeng, Ruyi Tan, Hongli Ma

**Affiliations:** Department of Anesthesiology, Chongqing University Cancer Hospital, Chongqing, China

**Keywords:** sleep disorders, malignant tumor, evidence summary, evidence-based nursing, postoperative management

## Abstract

**Introduction:**

This study aimed to extract, evaluate, and summarize the evidence related to the management of sleep disorders after malignant tumor surgery, providing a reference for evidence-based clinical practice.

**Methods:**

A systematic and hierarchical search strategy was used to identify relevant evidence from authoritative databases and resources. Following the “6S” evidence pyramid model, we conducted a comprehensive review of the following databases: BMJ Best Practice, CINAHL, Cochrane Library, Clinical Trials, Embase, PubMed, UpToDate, Web of Science, Guidelines International Network, National Guideline Clearinghouse, and the National Guidelines Database. Additionally, key institutional websites and specialized databases were consulted, such as the’ National Institute for Health and Care Excellence in the United Kingdom, ‘the Joanna Briggs Institute Centre for Evidence-Based Health Care in Australia, ‘the Registered Nurses’ Association of Ontario in Canada, the Scottish Intercollegiate Guidelines Network, China National Knowledge Infrastructure, China Biomedical Literature Database, Wan Fang Data, VIP Database, and Medlive. The search included clinical decisions, evidence summaries, guidelines, recommended practices, expert consensuses, systematic reviews, and randomized controlled trials. The retrieval period spanned from the inception of each database to December 31, 2024. Two researchers trained in evidence-based nursing independently evaluated the quality of the literature, extracted and synthesized the evidence, and incorporated expert recommendations as appropriate. This rigorous approach ensured comprehensive coverage of international and regional evidence-based resources, providing a solid foundation for our research.

**Results:**

Finally, we screened 12 articles with high-quality results (including 10 guidelines and 2 expert consensuses), providing 37 pieces of evidence covering four aspects: risk factors, evaluation methods, intervention measures, and effect evaluation after intervention.

**Discussion:**

The summarized evidence offers a reference for clinicians in managing sleep disorders in patients after malignant tumor surgery. However, the selection and application of evidence should be combined with specific circumstances to improve the postoperative rehabilitation of patients with malignant tumors.

## 1 Introduction

Sleep is a fundamental physiological requirement for human survival, essential for maintaining immune function, cognitive performance, emotional regulation, and physical recovery (1). Sustaining optimal sleep quality is particularly important during periods of physiological stress, such as surgery or illness.

Sleep disorders comprise a group of conditions characterized by disruptions in sleep quantity, quality, timing, or related behaviors that result in daytime impairment or distress (2). These conditions can be either acute or chronic and are typically categorized into insomnia, hypersomnia, circadian rhythm sleep–wake disorders, and sleep-related movement or respiratory disorders (2–4). According to the International Classification of Sleep Disorders, Third Edition (ICSD-3), acute sleep disturbances are often triggered by identifiable stressors and generally resolve within 3 months. In contrast, chronic disturbances persist beyond this period and are frequently associated with maladaptive cognitive and behavioral responses (5, 6).

Among patients with malignant tumors, sleep disturbances are highly prevalent and may be exacerbated by surgery, chemotherapy, radiotherapy, and the hospital environment (7). Insomnia is the most frequently reported subtype, particularly during the postoperative period. Contributing factors include pain, opioid use, environmental stimuli such as noise and light, and insufficient clinical attention to sleep-related issues (8–10). Studies have demonstrated that the prevalence of sleep disturbances varies depending on the type of surgery and the underlying malignancy (8, 9).

The pathophysiology of postoperative sleep disturbances is multifactorial. Biologically, surgical stress and sleep deprivation can trigger inflammatory responses, resulting in elevated levels of cytokines such as IL-1β, TNF-*α*, and IL-6, which negatively affect sleep quality (6, 11). Psychologically, factors such as anxiety, depression, and maladaptive coping mechanisms further contribute to the persistence of insomnia (5, 6). The “3-P” model—comprising predisposing, precipitating, and perpetuating factors—illustrates the complex interplay between biological vulnerability, perioperative stressors, and behavioral maladaptation in the development of chronic insomnia.

Both acute and chronic sleep disturbances after surgery have been associated with adverse clinical outcomes, including increased pain perception, delayed recovery, neurological complications, emotional distress, and reduced patient satisfaction (1, 12, 13). These consequences underscore the importance of early identification and targeted management of sleep disorders—particularly insomnia—in postoperative cancer care.

Nevertheless, the existing evidence on the management of postoperative sleep disturbances in cancer patients remains fragmented. Most available studies are observational in nature, involve small sample sizes, and exhibit considerable variability in assessment tools and intervention strategies. This inconsistency hinders the development of standardized clinical protocols and evidence-based recommendations.

Comprehensive and systematically developed evidence summaries are therefore urgently needed to support clinical decision-making and to establish a robust, unified foundation for practice. This study aimed to compile one of the most comprehensive available evidence on the management of sleep disorders following malignant tumor surgery. This study was registered through the Evidence-Based Nursing Center of Fudan University (registration number: ES20257203).

## 2 Materials and methods

### 2.1 Problem establishment

We employed the PIPOST tool developed by the Evidence-Based Nursing Center of Fudan University to construct our evidence-based question. P (Population): Patients diagnosed with malignant tumors undergoing surgery; I (Intervention): Management strategies for postoperative sleep disorders, including assessment and intervention; P (Professional): Physicians and nurses who implement the evidence; O (Outcome): Improvement of sleep quality and related symptoms; S (Setting): Clinical inpatient settings; T (Type of evidence): Clinical guidelines, expert consensuses, and systematic reviews. This structured approach ensured that our evidence synthesis remained patient-centered and clinically applicable (42, 43).

### 2.2 Evidence retrieval strategy

Following the “6S” evidence resource pyramid model (14), a structured and systematic search was conducted across multiple authoritative databases and resources, adhering to a hierarchical approach. The search encompassed the BMJ Best Practice, CINAHL, Cochrane Library, ClinicalTrials.gov, Embase, PubMed, UpToDate, Web of Science, Guidelines International Network, National Guideline Clearinghouse, and National Guidelines Database. Additionally, institutional archives and specialized databases were reviewed, including materials from the National Institute for Health and Care Excellence in the United Kingdom, Joanna Briggs Institute (JBI) Centre for Evidence-Based Health Care in Australia, Registered Nurses’ Association of Ontario in Canada, and the Scottish Intercollegiate Guidelines Network. For Chinese sources, we reviewed the China National Knowledge Infrastructure, China Biomedical Literature Database, Wanfang Data, VIP Database, and Medlive to ensure comprehensive coverage. The search period extended from the establishment of the database to December 31, 2024 (Table 1). When searching domestic and international websites or databases, the Chinese search terms included “Sleep disorders/insomnia, tumors/cancer, and postoperative,” whereas the English search terms were “neoplasms/cancer/oncology/tumor, sleep initiation and maintenance disorders/insomnia,” and “initiation and maintenance disorders/insomnia/sleep disorder/sleep disorders/sleep disturbance, postoperative.” Taking PubMed as an example, combination of subject terms and free-text words, the search formula: ((neoplasms OR cancer OR oncology OR tumor) AND ((sleep initiation and maintenance disorders OR insomnia OR sleep disorder OR sleep disorders OR sleep disturbance) AND postoperative)). To ensure a comprehensive and inclusive retrieval of evidence, the term “sleep disorders” was defined broadly to encompass any condition or symptom involving disturbances in sleep initiation, maintenance, duration, quality, or circadian regulation that result in daytime dysfunction. This included, but was not limited to, insomnia, poor sleep quality, sleep fragmentation, excessive daytime sleepiness, and circadian rhythm disruption (2). We adopted this inclusive approach to capture diverse clinical terminologies used in practice and across different guidelines, rather than relying solely on one diagnostic classification system.

**Table 1:** Literature search strategies.

Database	Key words or related words	Boolean logic	Time range
BMJ/CINAHL/Cochrane Library/Clinical Trials/PubMed/UpToDate/Web of Science	Neoplasms/cancer/oncology/tumor sleep disorder/sleep initiation and maintenance disorders/sleep disorders/sleep disturbance/insomnia postoperative	((neoplasms OR cancer OR oncology OR tumor) AND ((sleep initiation and maintenance disorders OR insomnia OR sleep disorder OR sleep disorders OR sleep disturbance) AND postoperative))	Inception—Dec 2024
Embase	Neoplasms/cancer/oncology/tumor sleep disorder/sleep initiation and maintenance disorders/sleep disorders/sleep disturbance/insomnia postoperative	(‘neoplasms’:ab,ti OR’cancer’:ab,ti OR’oncology’:ab,ti OR’tumor’:ab,ti) AND (‘sleep initiation and maintenance disorders’:ab,ti OR’insomnia’:ab,ti OR’sleep disorder’:ab,ti OR’sleep disorders’:ab,ti OR’sleep disturbance’:ab,ti) AND (“postoperative”:ab,ti)	
GIN/NGC/NICE/JBI/RNAO/SIGN	Sleep disorder/sleep initiation and maintenance disorders/sleep disorders/sleep disturbance/insomnia	sleep disorder/sleep initiation and maintenance disorders/sleep disorders/sleep disturbance/insomnia	
CNKI/CBM/VIP	Sleep disorders/Insomnia Tumors/Cancer Postoperative	(Sleep disorders OR Insomnia) AND (Tumors OR Cancer) AND Postoperative (In Chinese)	
WFD	Sleep disorders/Insomnia Tumors/Cancer Postoperative	((Sleep disorders | Insomnia) + (Tumors | Cancer)) + Postoperative (In Chinese)	
Medlive	Sleep disorders/Insomnia/Sleep quality	Sleep disorders/Insomnia/Sleep quality (In Chinese)	

### 2.3 Inclusion and exclusion criteria

#### 2.3.1 Inclusion criteria

1. Studies focusing on patients with malignant tumors who underwent surgical intervention.
2. Evidence pertaining to the recognition of risk factors, evaluation techniques, management strategies, and assessment of their impact on postoperative sleep disturbances in patients with malignant tumors.
3. Acceptable categories of evidence encompass clinical practice guidelines, expert consensus documents, evidence synopses, and systematic reviews.

#### 2.3.2 Exclusion criteria

(1) Literature that has been repeatedly published or updated; (2) studies with incomplete or inaccessible data; (3) Publications of low quality based on established evaluation criteria; (4) Documents that are guideline interpretations or implementation plans.

### 2.4 Data extraction and synthesis strategy

Two researchers trained in evidence-based systems independently completed an assessment of the quality of the included literature based on predetermined inclusion and exclusion criteria. Their assessments were cross-verified, and discrepancies between the two evaluators were resolved through consultation with a third expert in evidence-based medicine. Two researchers who systematically studied the evidence-based nursing course evaluated the included literature and cross-checked it using appropriate evaluation tools. Any disagreements were resolved by a third expert in evidence-based medicine, who reached a consistent conclusion.

We used thematic analysis to identify and summarize recurring topics across the included guidelines and expert consensus documents. Four major themes were extracted: risk factors, assessment methods, intervention strategies, and evaluation.

The extracted content included: (1) evidence-based recommendations related to the management of sleep disorders following malignant tumor surgery; (2) classification of each recommendation into one of four domains—risk factors, assessment tools, intervention strategies, and outcome indicators; (3) the level and grading of evidence, if available; and (4) reported or expected outcomes such as improvements in sleep quality, reduction in insomnia symptoms, and enhancement of postoperative recovery (e.g., pain relief, psychological well-being, or fatigue reduction). In this review, “relevant evidence” is defined as any recommendation that addresses sleep-related disturbances—such as insomnia, fragmented sleep, reduced sleep quality, or circadian disruption—within the perioperative or cancer care setting. For synthesis, a thematic categorization approach was used. All extracted recommendations were grouped into the four main evidence domains. Within each domain, recommendations were further organized according to the type of intervention (e.g., cognitive, behavioral, pharmacological) or evaluation method. Overlapping evidence from multiple sources was consolidated, while unique recommendations were retained as supplementary guidance.

### 2.5 Evidence quality evaluation criteria

The quality of the guidelines was assessed using the AGREE II guideline Quality Assessment form (15). Agree II was revised in 2009 and updated in 2017 by the International Working Group for Research and Evaluation of Clinical Guidelines, comprised researchers from 13 countries, including Canada and the United Kingdom. This tool builds on the AGREE issued in 2003. The AGREE II comprises 23 items across six domains, with each item rated on a seven-point scale (1 = strong disagreement and 7 = strong agreement). The standardized score for each domain was calculated using the formula: [(actual score − minimum possible score) / (maximum possible score − minimum possible score)] × 100. All domain scores were evaluated collectively, and a unified threshold for the six domains was established through expert consensus. Guidelines received a quality rating of A if the standardized score in each domain was 70% or higher. If the standardized score ranged between 30 and 70%, the guideline quality was rated as B. To further enhance clarity and reduce redundancy, the quality rating criteria were reorganized as follows: guidelines earned an A rating when all six domains achieved a standardized score of at least 70%. Conversely, guidelines with a standardized score within the range of 30–70% in any domain were assigned a B rating. In this summary of evidence, the literature that failed to reach the level of grade B or above has been excluded.

The quality of the included guidelines was assessed using the JBI Center for Evidence-Based Health Care Expert Consensus Evaluation Criteria (2017 edition), which consists of six items and employs a grading system of “yes,” “no,” “unclear,” and “not applicable” (16). For systematic evaluation, the 2014 edition of the JBI Evidence-Based Practice Center Standards was used. This tool includes 11 items, each evaluated based on the criteria of “yes,” “no,” “unclear,” or “not applicable” (17). The evidence was synthesized by tracing back to the original source documents, and the appropriate JBI Center for Evidence-Based Health Care (2016 edition) tool was selected for quality assessment based on literature type.

Calculate the proportion of “Yes” responses and determine the overall rating based on the following criteria:

High quality (≥5 items rated “Yes”): The guideline meets quality standards in most key areas and is recommended for inclusion in the analysis.
Moderate quality (3–4 items rated “Yes”): The guideline meets quality standards in some areas but may have certain limitations, requiring cautious interpretation.
Low quality (≤2 items rated “Yes”): The guideline does not meet quality standards in most areas and is not recommended for inclusion in the analysis.

## 3 Results

### 3.1 Literature search and basic characteristics of the included literature

**Figure 1:**
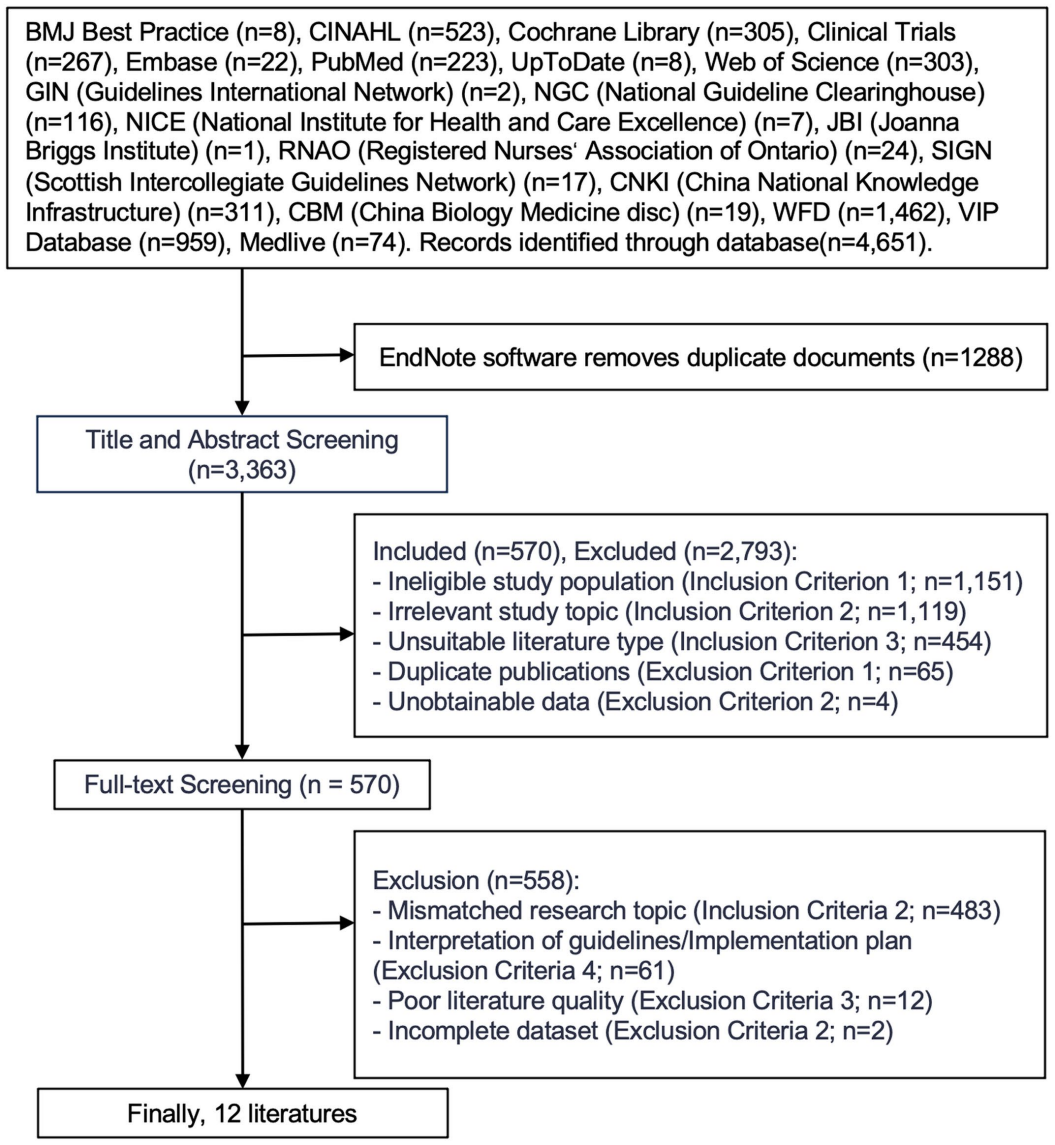
Flow chart of literature screening.

In total, 4,651 articles were initially identified through database searching. After removing duplicates, 3,363 articles remained. Following a review of titles and abstracts, 570 articles were selected for further screening. After a full-text review based on the inclusion and exclusion criteria, 12 articles were finally included in this review, consisting of 10 clinical practice guidelines and 2 expert consensus statements. The literature screening process is illustrated in Figure 1, and the main characteristics of the included studies are summarized in Table 2.

**Table 2:** Characteristics of included studies (*n* = 12).

Included literature	Literature sources	Literature types	Literature themes	Publication year
Luciano Ferreira Drager et al. (18)	PubMed	Guideline	2023 Guidelines on the Diagnosis and Treatment of Insomnia in Adults – Brazilian Sleep Association	2023
Wai Ching Lam et al. (19)	PubMed	Guideline	Hong Kong Chinese Medicine Clinical Practice Guideline for Cancer Palliative Care: Pain, Constipation, and Insomnia	2019
Jack D. Edinger et al. (20)	PubMed	Guideline	Behavioral and psychological treatments for chronic insomnia disorder in adults: an American Academy of Sleep Medicine clinical practice guideline	2020
Cristina Frange et al. (21)	PubMed	Guideline	Practice recommendations for the role of physiotherapy in the management of sleep disorders: the 2022 Brazilian Sleep Association Guidelines	2022
Dieter Riemann et al. (23)	PubMed	Guideline	The European Insomnia Guideline: An update on the diagnosis and treatment of insomnia 2023	2023
Hayun Choi et al. (24)	PubMed	Guideline	Korean Clinical Practice Guideline for the Diagnosis and Treatment of Insomnia in Adults	2020
Michael Howell et al. (25)	PubMed	Guideline	Management of rapid eye movement (REM) sleep behavior disorder: an American Academy of Sleep Medicine clinical practice guideline	2023
Tara Sanft et al. (3)	PubMed	Guideline	Survivorship, Version 1. 2023 Featured Updates to the NCCN Guidelines	2023
Zhang jian jun et al. (26)	CNKI	Guideline	Clinical practice guidelines for cancer-related fatigue in China (2021 edition)	2021
Wang yu ping, et al. (27)	CNKI	Guideline	Chinese guideline for diagnosis and treatment of insomnia (2023)	2023
TangLili et al. (30)	CNKI	Expert Consensus	Adult cancer patients insomnia diagnosis and treatment expert advice	2021
Kun-Ming Rau et al. (22)	PubMed	Expert Consensus	Management of cancer-related fatigue in Taiwan: an evidence-based consensus for screening, assessment, and treatment.	2023

The included publications were issued between 2019 and 2023, with the majority published in 2023 (*n* = 5), reflecting the timeliness and clinical relevance of the evidence. In terms of source distribution, nine articles were retrieved from PubMed, and three from CNKI, representing a blend of international and Chinese literature. These documents cover a wide range of themes related to postoperative sleep disturbances in patients with malignant tumors, including the diagnosis and treatment of insomnia, cancer-related fatigue, REM sleep behavior disorder, and comprehensive survivorship care. They were issued by authoritative institutions such as the American Academy of Sleep Medicine, the Brazilian Sleep Association, the National Comprehensive Cancer Network (NCCN), and national traditional Chinese medicine organizations, offering a diverse and multidisciplinary perspective on the subject.

### 3.2 Quality evaluation results of the included articles

#### 3.2.1 Quality evaluation of clinical guidelines

Ten guidelines were included (3, 18–27). The evaluation results showed that the literature writing process was rigorous and the content was detailed, with a high overall quality, and all were eligible for inclusion (Table 3).

**Table 3:** Results of clinical guideline quality evaluation (*n* = 10).

Inclusion guidelines	Percentage of standardization in each field of the guideline (%)						The number of fields with a proportion of ≥ 60% (unit)	The number of fields with a proportion of ≥30% (unit)	Level
	Scope and purpose	Participants	Rigorousness of formulation	Clarity of expression	Applicability of the guidelines	Independence of compilation			
Luciano Ferreira Drager et al. (22)	88.9	69.4	65.6	91.7	64.6	70.8	6	6	A
Wai Ching Lam et al. (18)	97.2	88.9	100	100	60.4	75.0	6	6	A
Jack D. Edinger et al. (19)	88.9	64.4	63.1	83.3	66.3	45.8	5	6	B
Cristina Frange et al. (20)	100	86.1	79.2	100	91.6	91.7	6	6	A
Dieter Riemann et al. (23)	100	72.2	80.2	100	77.1	70.8	6	6	A
Hayun Cho, et al. (24)	97.2	88.9	80.2	97.2	91.7	75.0	6	6	A
Michael Howell et al. (25)	100	77.8	86.5	100	91.7	83.3	6	6	A
Zhang jian jun et al. (26)	91.7	917	70.8	94.4	45.8	54.2	5	6	B
Wang yu ping et al. (27)	100	100	89.6	100	95.8	95.8	6	6	A
Tara Sanft et al. (3)	88.9	72.2	63.5	75	47.9	33.3	4	6	B

#### 3.2.2 Quality evaluation of expert consensus

Two expert consensus documents were included (22, 27). Two expert consensus were evaluated using the JBI 2016 version of the expert consensus evaluation tool, and all six items were rated as “yes.” These expert consensuses had a high quality and were eligible for inclusion.

### 3.3 Evidence summary

A total of 12 studies were included in the initial draft of the evidence summary (3, 18–27), from which 37 best-evidence statements were ultimately synthesized. The evidence spans four major domains: risk factors, assessment methods, intervention strategies, and evaluation of intervention outcomes. Table 4 provides a detailed breakdown of the extracted evidence. The levels of evidence were classified as follows: 1a-meta-analysis of homogeneous randomized controlled trials (RCTs); 1b-a single, well-designed RCT; 2a-prospective cohort study; 2b-retrospective cohort study or low-quality prospective study; and 4b-single-center or small-sample cross-sectional study.

**Table 4:** Evidence summary for the management of sleep disorders after malignant tumor surgery.

Categories	Content of evidence	Level of evidence
Risk factors	The cancer itself and its treatment	
	Cancer factors: different types of cancer have an impact on the incidence of CRF. The incidence of CRF in patients with lung cancer is relatively high, up to 70–100%. Various cancers exhibit distinct biological characteristics, treatment approaches, and impacts on the body, potentially influencing sleep patterns indirectly (26).	1a
	Surgical trauma: Surgical trauma can cause an inflammatory response that can lead to changes in hormone levels, cause pain and discomfort, and interfere with sleep (18–23, 26, 27).	1a
	Type of anesthesia: General anesthesia may cause transient postoperative loss of consciousness and respiratory depression, affecting sleep coherence; Local anesthesia has minimal impact on overall sleep quality (19).	2a
	Pharmacological factors: Certain medications, such as selective serotonin reuptake inhibitors, have been shown to induce Rapid eye movement (REM) sleep behavior disorder (RBD). This finding indicates that the administration of drugs that can affect sleep patterns, including opioid analgesics and specific psychotropic agents, may contribute to post-surgical sleep disturbances in patients with malignant tumors (25).	2b
	Chemoradiotherapy side effects: nausea, vomiting, fatigue, and other discomfort affect sleep quality (19–23, 26).	2a
	Complications: Postoperative complications such as infection, bleeding, and atelectasis can affect sleep, increase psychological burden, and aggravate sleep disorders (20, 26, 27).	2b
	Psychological factors	
	Anxiety and depression: As a stressful event, surgery easily causes anxiety and depression in patients, which can affect the function of the nervous system. Patients’ concerns about surgical outcome, such as tumor recurrence and metastasis, will increase psychological stress, interfere with sleep, and lead to sleep disorders (3, 18–24, 26, 27).	1a
	Nutritional status	
	Post-surgical patients with malignant tumors frequently experience a decline in nutritional status, and malnutrition can impair physical recovery, resulting in persistent fatigue, which subsequently affects sleep quality (26).	2b
	Environmental factors	
	Hospital-related factors such as noise and lighting can disrupt sleep patterns. Unfamiliar environment in hospital wards, changes in sleep habits, frequent medical operations, and noise of medical equipment may lead to sleep disorders (3, 18–24, 27).	2b
	Age factor	
	Age serves as a significant risk factor for RBD, with older adults being more prone to developing RBD. This finding implies that elderly patients undergoing malignant tumor surgery may have an increased susceptibility to post-surgical sleep disturbances (25).	1a
	Socio-demographic factors	
	Elderly patients and women with malignant tumors who undergo surgical procedures are more susceptible to sleep disturbances (25, 30).	1a
	Neurodegenerative diseases	
	REM sleep behavior disorder is commonly observed in conjunction with neurodegenerative conditions such as Parkinson’s disease and dementia with Lewy bodies. Postoperative neurological complications or potential neuropathy in patients with malignant tumors may heighten the risk of developing sleep disorders (25).	1a
Methods of assessment	Clinical evaluation	
	Medical history collection and physical examination: A comprehensive evaluation of the patient’s medical history, including detailed inquiries into sleep patterns, pain levels, and psychological state, in conjunction with a thorough physical examination, provides critical information on the patient’s overall health condition and serves as a foundation for accurate diagnosis (18–27).	1a
	Subjective assessment	
	Sleep diary Maintaining a sleep diary that records key sleep-related data such as bedtime, wake-up time, and frequency of awakenings, offers valuable insights into the patient’s sleep quality and is an essential tool for assessing sleep disorders (22).	1a
	Subjective scale assessment Using standardized scales like the Pittsburgh Sleep Quality Index (PSQI), Insomnia Severity Index (ISI), Athens Insomnia Scale, Hamilton Anxiety Rating Scale (HAMA), and Hamilton Depression Rating Scale (HAMD) allows for a thorough evaluation of sleep quality, insomnia severity, and emotional status. These tools provide objective measures of the patient’s sleep and psychological well-being (19–25).	1a
	Based on Traditional Chinese Medicine principles, patients are evaluated to determine their specific syndrome type, such as liver qi stagnation, spleen deficiency with liver qi stagnation, qi and blood deficiency, qi and yin deficiency, or heart and spleen deficiency. This differentiation guides the development of personalized treatment plans (18).	4b
	Objective assessment	
	Polysomnography (PSG), considered the gold standard for diagnosing sleep disorders, accurately assesses sleep architecture, respiratory function, and other relevant parameters, providing crucial data for diagnosis and efficacy evaluation (18–27).	1a
	Actigraphy: Actigraphy monitors sleep–wake cycles and activity levels, offering supplementary data that aids in assessing sleep patterns (27).	2b
Interventions	Non-pharmacological treatments	
	Sleep hygiene education: guiding patients in developing healthy sleep habits, such as maintaining a regular schedule for work and rest, creating a comfortable sleep environment, avoiding stimulating activities before bed, and minimizing medical interventions prior to sleep, is crucial for enhancing sleep quality (20, 21).	1a
	Cognitive behavioral therapy (CBT-I) This therapeutic approach encompasses techniques such as sleep restriction, stimulus control, cognitive restructuring, and relaxation training, all of which can effectively address patients’ sleep-related cognitions and behaviors, thereby improving sleep disorders. Forms of treatment Face-to-face: Standard CBT-I model, personalized treatment, good efficacy, but higher cost. Digital network model: leveraging intelligent devices and specialized applications, this approach is not constrained by location and offers cost-effectiveness. However, it faces challenges with patient adherence and lacks personalized elements (19–25).	1a
	Relaxation training: methods like deep breathing exercises, progressive muscle relaxation, and mindfulness meditation are frequently utilized to alleviate patient tension and anxiety, thereby promoting better sleep (19).	2b
	Sound therapy Music therapy demonstrates efficacy in reducing sleep onset latency, enhancing sleep efficiency and quality. However, standardized protocol for music intervention regarding program selection, timing, frequency, or duration is lacking (24). Closed-loop pink noise stimulation during sleep, delivered via a pink noise simulator, can enhance slow wave activity and increase slow wave oscillations without inducing arousal, thereby improving sleep quality. However, its efficacy in treating insomnia requires further investigation (27).	1a 2b
	Light therapy: Light therapy as a natural and simple treatment, especially morning phototherapy, can improve sleep quality and increase sleep maintenance by adjusting the endogenous sleep–wake cycle, but the effect size is small to moderate, and additional research is needed to demonstrate clinical benefits in chronic insomnia.	2b
	Physical therapy	
	Transcranial magnetic stimulation (TMS): Repetitive TMS (rTMS) has the strongest evidence base. Low-frequency (≤1 Hz) TMS can reduce cortical excitability, serving as a safe and effective treatment for chronic insomnia. It can be used as a standalone therapy or in combination with other treatments. In China, applying low-frequency stimulation to the bilateral dorsolateral prefrontal cortex and parieto-occipital regions is recommended to enhance sleep quality (27).	1b
	Transcranial electrical stimulation: Transcranial direct current stimulation (tDCS) and transcranial alternating current stimulation (tACS) show promise in the treatment of insomnia. Small-sample studies on tDCS have demonstrated its safety, efficacy, and tolerability, with improvements in sleep quality. Moreover, tACS technology can enhance the amplitude of slow wave oscillations, thereby deepening sleep (27).	1b
	Medication	
	Dexmedetomidine: The administration of dexmedetomidine during the anesthesia period may help prevent the development of postoperative sleep disorders (27).	1a
	Benzodiazepine (BZD) agonists: BZDs such as zolpidem and dexzopiclone can improve the symptoms of insomnia, but drug dependence and adverse reactions should be monitored. BZDs should be used in patients with liver and kidney dysfunction, myasthenia gravis, dementia with Lewy bodies, and moderate-to-severe obstructive sleep apnea (27).	1a
	Melatonin receptor agonists: For example, ramelamide and agomelatine can shorten sleep latency, improve sleep efficiency, and increase total sleep time. They are used for insomnia caused by circadian rhythm disorder with difficulty falling asleep as the main manifestation. They have no dependence and addiction, no withdrawal symptoms, no respiratory depression, and little residual effect on the next day (26, 27).	2a
	Dual orexin receptor antagonists: Suvaresan, lebolesen, and daliresan can be adjusted to significantly improve sleep efficiency, shorten sleep latency, and reduce wake time after sleep, with good safety and tolerance (27).	1a,b
	Antihistamine H1 receptor drugs: Doxylamine has sedative effect, can shorten sleep latency, and is used for treating acute insomnia (27).	1b
	Antidepressants: Antidepressants such as doxepin and trazodone have sedative effects and can improve insomnia symptoms, especially for patients with anxiety and depressive symptoms (22–24, 27).	2b
Effect evaluation	Evaluation of effectiveness	
	Sleep quality measures	
	Changes of PSG: Many studies have discovered that sleep latency was shortened, sleep efficiency was improved, and REM sleep and slow wave sleep returned to normal after the intervention of postoperative patients (20–27).	1a
	Subjective rating scale scores	
	For example, the patient’s PSQI score was significantly reduced, indicating that sleep quality and insomnia severity were improved (19–25).	1a
	Daytime functional improvement	
	Reduced fatigue: As assessed using the FSS and other scales, patients’ fatigue symptoms were reduced, their physical strength and energy were restored, and they were better able to perform daily activities (26).	1a
	Improved emotional state	
	Anxiety and depression were relieved The scores of HAMA and HAMD scales were decreased, the anxiety and depression of patients were alleviated, and the emotional stability was improved (27).	1a
	Safety evaluation	
	Adverse drug reactions: closely observe the adverse drug reactions, such as somnolence, dizziness, unsteady gait of sedative and hypnotic drugs, and the adverse drug reactions of antidepressant drugs. Studies have discovered that the rational use of drugs can reduce the occurrence of adverse reactions (27).	1a

1a, Meta-analysis of homogeneous randomized controlled trials; 1b, A single well-designed RCT study; 2a, Prospective cohort study; 2b, Retrospective cohort study or low-quality prospective study; 4b, Cross-sectional study conducted in a single center or with a small sample size.

Within the domain of risk factors, the literature identified both biological and psychosocial contributors to postoperative sleep disturbances in patients with malignant tumors (18–21). Key biological and treatment-related factors included cancer type, surgical trauma, anesthesia technique, pharmacological side effects (e.g., opioids, selective serotonin reuptake inhibitors), complications, and side effects of chemo-or radiotherapy. Psychosocial and environmental contributors such as anxiety, depression, nutritional status, hospital environment, and age-related vulnerability—particularly to REM sleep behavior disorder—were also frequently highlighted (18, 20, 22, 23). The level of supporting evidence for these factors ranged from 1a to 2b.

Assessment methods were divided into subjective and objective approaches. Subjective tools included sleep diaries and standardized instruments such as the Pittsburgh Sleep Quality Index (PSQI), Insomnia Severity Index (ISI), Hamilton Anxiety Scale (HAMA), and Hamilton Depression Scale (HAMD) (18, 19, 23). Objective assessments primarily included polysomnography (PSG)—regarded as the gold standard—and actigraphy (21, 24). In Chinese clinical guidelines, Traditional Chinese Medicine (TCM) syndrome differentiation was also used to guide personalized assessment and treatment (26).

Intervention strategies involved a combination of non-pharmacological and pharmacological approaches. Non-pharmacological treatments included cognitive behavioral therapy for insomnia (CBT-I), relaxation training, music therapy, and light therapy (19, 21, 22, 25). Physical therapies such as transcranial magnetic stimulation (TMS) and transcranial electrical stimulation (tDCS, tACS) were also reported (22, 25, 27). Pharmacological interventions included dexmedetomidine, benzodiazepine receptor agonists, melatonin receptor agonists, dual orexin receptor antagonists, antihistamines, and antidepressants (20, 21, 23).

The effectiveness of these interventions was evaluated based on improvements in PSG parameters, reductions in subjective sleep and emotional scores, relief of fatigue symptoms, and enhancement of daytime functioning (18, 22, 23, 25). Additionally, several studies emphasized the importance of monitoring adverse drug reactions to ensure treatment safety (20, 21).

In summary, the included studies provide comprehensive, high-quality, and multidisciplinary evidence for the assessment and management of sleep disturbances in postoperative cancer patients, thereby supporting evidence-based clinical decision-making (3, 18–27).

## 4 Discussion

### 4.1 Evidence synthesis process

This study adhered to stringent scientific standards, yielding high-quality evidence to guide clinical nursing practices. All research team members received comprehensive training in evidence-based nursing methodologies. Two researchers independently conducted evidence retrieval, quality assessment, data extraction, and evidence grading to ensure objectivity and reliability. Furthermore, sleep specialists oversaw quality control and reviewed the evidence to maintain scientific rigor of the synthesis process. Two expert consensuses were included, meeting all quality criteria. Ten guidelines were also included, and international peers rigorously reviewed and validated the evidence, ensuring their robustness and credibility (3, 18–27). These studies addressed risk factors, assessment methods, intervention strategies, and evaluation of sleep disorders following malignant tumor surgery. The research team critically evaluated the strengths and limitations of the evidence while considering the clinical context. Through a comprehensive analysis and synthesis, 37 optimal pieces of evidence were identified for managing postoperative sleep disorders. This evidence provides precise and scientifically sound guidance for enhancing the management of post-surgical sleep disorders, ultimately improving patient care and sleep quality. These four themes provide a structured and comprehensive overview of the current evidence and practical recommendations, contributing to a clearer understanding of postoperative sleep disorder management.

### 4.2 Early identification of risk factors for postoperative sleep disorders in patients with malignant tumors is essential

Postoperative sleep disturbance in patients with cancer arise from multiply risk factors. Surgical intervention, chemotherapy, radiotherapy, and multiple therapeutic modalities can contribute to the onset and worsening of sleep disorders over time (7). Certain adjuvant medications may also impact sleep patterns; however, their influence generally diminishes as treatment progresses (28). Psychological factors, such as pre-existing anxiety and depression, significantly impair sleep quality, particularly if these conditions are present before the initiation of cancer treatment. Implementing emotion regulation strategies can help mitigate sleep disturbances (29). Regarding sleep-related cognition and behavior, negative thought patterns, including excessive worry before bedtime and catastrophic thinking, can lead to chronic insomnia (30, 31). Demographic characteristics also exert a substantial influence; with women, younger individuals, unemployed populations, and those from lower-income households being more susceptible. The impact of educational attainment on sleep outcomes remains inconsistent, potentially owing to variations in study populations, sample sizes, and treatment methods. Disease-specific factors and pre-treatment health status are critical determinants of sleep quality. More advanced disease stages were correlated with more severe sleep disturbances. Additional contributing factors include regional lymph node metastasis, pretreatment use of analgesics, higher number of comorbidities, poorer functional status, elevated body mass index, and preexisting sleep issues (29). Given the complexity of these risk factors, healthcare providers should leverage the evidence presented here for early identification. Targeted preventive measures should be implemented to address modifiable risk factors and enhance patient wellbeing.

### 4.3 Comprehensive assessment through subjective and objective evaluations

Evaluating sleep disorders encompass subjective and objective evaluations. Currently, most studies use subjective scales for evaluation. Among the various assessment tools, the Pittsburgh sleep quality index (PSQI) developed by Buysse et al. (32) is the most widely utilized internationally and domestically. Additionally, Lee’s general sleep disturbance scale (GSDS) is frequently employed and has demonstrated robust reliability and validity in cancer patient populations (33). Unlike the PSQI, the GSDS employs an 8-point scoring system that allows it to detect changes across different sleep factors more sensitively. Several studies have utilized the insomnia severity index and the Athens insomnia scale to assess insomnia. The insomnia severity index consists of seven items and focuses on assessing the severity of insomnia, emphasizing its adverse psychological impact. It has shown strong reliability and validity in patients with malignant tumors. The Athens insomnia scale, which includes eight items, primarily evaluates sleep quality, duration, and daytime dysfunction caused by insomnia (34). Its concise and practical nature enables accurate identification of insomnia cases. Polysomnography (PSG) provides comprehensive sleep measurements and is critical for diagnosing complex sleep disorders. However, PSG is generally unnecessary for evaluating insomnia symptoms and is unsuitable for the routine assessment of chronic insomnia. PSG should be considered in pathological sleepiness or report other sleep-related pathologies, such as sleep breathing disorders, periodic limb movements, or parasomnias (35). Some studies have combined objective measurement methods such as wrist actigraphy with subjective assessments using the PSQI to dynamically monitor patients’ sleep conditions and gather more comprehensive and objective data. However, most studies rely solely on subjective scales because of time and financial constraints, often lacking integration with objective assessments such as measurement instruments and biochemical indicators. Future research should adopt a combined approach of subjective and objective assessments (36). Initially, subjective scales were used to evaluate patients. If multiple pieces of evidence suggest the presence of related sleep disorders, objective assessments such as PSG should be employed to reflect the dynamic changes in sleep disorders more accurately.

### 4.4 Key considerations for developing effective intervention measures

Intervention strategies for postoperative sleep disorders in patients with malignant tumors can be broadly categorized into non-pharmacological and pharmacological approaches. Additionally, global consensus on which pharmacological treatment provides optimal efficacy or the best risk–benefit ratio is lacking. Cognitive behavioral therapy for Insomnia (CBT-I) is widely recognized as the first-line treatment (37). CBT-I and pharmacological interventions may produce comparable short-term outcomes; however, only CBT-I has demonstrated sustained long-term benefits post-treatment. Combining CBT-I with medication can accelerate the initial treatment response but may compromise the long-lasting positive effects of CBT-I (38). In specific cases, alternative interventions such as the use of dexmedetomidine during surgery, music therapy, traditional Chinese medicine, and physical rehabilitation exercises have shown efficacy in managing sleep disorders (27, 39). Following clinical assessment, when patients are in the early stages of sleep disorders, priority should be given to implementing psychological care interventions. If symptoms remain unalleviated, pharmacological treatment may then be introduced as an adjunctive measure for symptom control. When resources allow, cranial nerve stimulation therapy or a comprehensive treatment approach can be considered. Anesthesiologists must systematically refine perioperative pain management strategies to mitigate the risk of postoperative sleep disturbances. Psychological counseling should be integrated into routine postoperative care protocols as a key component of psychological intervention. Moreover, a systematic and standardized framework for managing sleep disorders should be developed, leveraging multidisciplinary expertise from fields such as anesthesiology, psychiatry, and nursing to foster interdisciplinary collaboration. A comprehensive approach should consider the patient’s medical condition, personal preferences, and available treatment resources to develop an intervention plan for postoperative sleep disorders in patients with malignant tumors.

### 4.5 Limitations

Despite considerable progress in the study of sleep disorders following malignant tumor surgery, several limitations remain. One of the primary challenges is the insufficient understanding of the complex interactions among various risk factors. In particular, the interplay between psychological and physiological elements—such as the inflammatory response induced by surgical trauma and psychological stress—has not been thoroughly elucidated (2, 28, 40). Further research is warranted to clarify these interrelationships, which may inform the development of more targeted and effective therapeutic strategies.

Cultural and regional differences in the management of sleep disorders also represent a critical area of concern. Variability in healthcare infrastructure, sociocultural attitudes, and accessibility of treatment may influence both the assessment and intervention approaches employed in different populations. Although the integration of multiple assessment modalities can enhance diagnostic accuracy, the validity and applicability of certain tools may vary across cultural and demographic contexts (19, 23, 25, 38, 39). Consequently, there is a pressing need to develop culturally sensitive, efficient, and clinically applicable assessment instruments that are adapted to specific populations, disease profiles, and healthcare workflows.

With regard to intervention strategies, novel therapies such as transcranial alternating current stimulation and pink noise stimulation have shown preliminary promise in enhancing sleep quality. However, existing studies are constrained by limited sample sizes and a lack of large-scale, high-quality clinical trials. The long-term efficacy and safety profiles of these interventions remain uncertain and must be substantiated through further rigorous investigation before their integration into routine clinical practice can be recommended (11, 18, 41).

This review provides a comprehensive synthesis of the current evidence concerning risk factors, diagnostic tools, therapeutic interventions, and treatment outcomes associated with postoperative sleep disorders in patients with malignant tumors. Nonetheless, future research is essential to establish more robust and cohesive links among these domains, thereby improving clinical relevance and informing optimized, patient-centered care strategies.

## 5 Conclusion

This evidence summary integrates 37 high-quality studies on the management of postoperative sleep disorders in patients with malignant tumors, emphasizing the multifactorial complexity of sleep disturbances in this population. The interplay of biological factors such as surgical trauma and anesthesia effects, along with socio-psychological factors like opioid use and emotional distress, significantly elevates the risk of sleep disorders. Accurate assessment necessitates a combination of subjective scales and objective monitoring tools. Additionally, traditional Chinese medicine syndrome differentiation can facilitate individualized diagnosis. Non-pharmacological interventions, including cognitive behavioral therapy, music therapy, and light therapy, have been demonstrated to be safe and effective primary options, while pharmacological and complementary therapies serve as supplementary approaches. Effectiveness evaluation should encompass multiple dimensions, such as sleep quality, emotional well-being, and functional recovery. Future clinical practice should consider patient preferences and resource availability, promote multidisciplinary collaboration, optimize treatment strategies, and ultimately enhance patient quality of life and prognosis.

## Data Availability

The data analyzed in this study is subject to the following licenses/restrictions: this article aims to systematically summarize and synthesize the evidence presented in systematic reviews, clinical practice guidelines, expert consensus statements, and related literature. The current query is not applicable to this context. Requests to access these datasets should be directed to YL,15923295225@163.com.
